# Oncogenic Events Dictate the Types and Locations of Gynecological Malignancies Originating from *Krt8*^+^ Mesothelial and Müllerian-Derived Epithelial Cells

**DOI:** 10.3390/cancers14030841

**Published:** 2022-02-08

**Authors:** Eun-Sil Park, Dongxi Xiang, Ying Xie, Roderick T. Bronson, Zhe Li

**Affiliations:** 1 Division of Genetics, Brigham and Women’s Hospital, Boston, MA 02115, USA; eunsil.park@merck.com (E.-S.P.); dxiang@shsmu.edu.cn (D.X.); sherryyx36@163.com (Y.X.); 2 Department of Medicine, Harvard Medical School, Boston, MA 02115, USA; 3 Rodent Histopathology, Harvard Medical School, Boston, MA 02115, USA; roderick_bronson@hms.harvard.edu

**Keywords:** ovarian cancer, uterine cancer, mouse modeling, cellular origin, Keratin 8 (K8), Müllerian-derived epithelial cells, mesothelial cells, ovarian surface epithelial (OSE) cells, fallopian tube epithelial (FTE) cells, endometrial cells

## Abstract

**Simple Summary:**

Ovarian and uterine cancers are the most common gynecological malignancies in women. The early detection, prevention, and treatment of these gynecological cancers can benefit from a better understanding of how tumor-initiating cells in them are formed from their corresponding target cell populations in the female reproductive system. To study this, we utilized a genetic approach in mice to introduce driver mutations commonly found in these cancers to Keratin 8 positive (K8^+^) mesothelial and epithelial cells in the ovary, fallopian tube, and uterus. We found that p53-loss appears to preferentially affect K8^+^ epithelial cells, leading to the development of uterine and ovarian malignancies, whereas PTEN-loss may preferentially affect mesothelial cells, leading to the development of ovarian endometrioid malignancies or adenoma on the fallopian tube surface. Collectively, our data suggest that oncogenic driver mutations may dominantly determine the locations and types of gynecological malignancies developed from K8^+^ mesothelial and epithelial cells in the female reproductive system.

**Abstract:**

Ovarian and uterine cancers are the most prevalent types of gynecological malignancies originating from mesothelial and/or Müllerian-derived epithelial cells. Recent genomic studies have identified common mutations in them that affect signaling pathways such as p53, PTEN/PI3K, RAS, and WNT pathways. However, how these mutations and their corresponding deregulated pathways affect gynecological cancer development from their cells-of-origin remains largely elusive. To address this, we performed the intrabursal injection of Cre-expressing adenovirus under the control of *Krt8* promoter (*Ad-K8-Cre*) to mice carrying combinations of various conditional alleles for cancer genes. We found that *Ad-K8-Cre* specifically targeted mesothelial cells, including ovarian surface epithelial (OSE) cells (mainly the LGR5^+^ subset of OSE cells) and mesothelial cells lining the fallopian tube (FT) serosa; the injected *Ad-K8-Cre* also targeted Müllerian-derived epithelial cells, including FT epithelial cells and uterine endometrial epithelial cells. The loss of p53 may preferentially affect Müllerian-derived epithelial cells, leading to the development of uterine and ovarian malignancies, whereas PTEN-loss may preferentially affect mesothelial cells, leading to the development of ovarian endometrioid malignancies (upon KRAS-activation or APC-loss) or adenoma on the FT surface (upon DICER-loss). Overall, our data suggest that different *Krt8*^+^ mesothelial and epithelial cell types in the female reproductive system may have different sensitivities toward oncogenic mutations and, as a result, oncogenic events may dominantly determine the locations and types of the gynecological malignancies developed from them.

## 1. Introduction

Among gynecological cancers, uterine cancer is the most common type. In the United States, the American Cancer Society estimated that in 2019, ~61,880 new cases of uterine cancers would be diagnosed and ~12,160 women would die from this cancer. Ovarian cancer is another common type of gynecological cancer and also the deadliest gynecological disease in women, with an overall 5 year survival rate of only ~30–46% [1]. Despite the high mortality, the early detection of ovarian cancer remains challenging by current screening technology due to the lack of specific biomarkers [2,3,4]. Both uterine and ovarian cancers are heterogeneous and are traditionally classified into several histologic subtypes, such as the common serous and endometrioid subtypes, as well as the rarer carcinosarcoma and sarcoma subtypes [5,6,7,8]. Recent large-scale genomic sequencing studies defined the mutational landscapes of these gynecological malignancies. Integrated genomic studies of uterine cancer revealed that uterine serous tumors and high-grade endometrioid tumors have extensive copy number alterations and frequent *TP53* mutations, whereas most endometrioid tumors have frequent mutations in the PTEN/PI3K, WNT, RAS, and SWI/SNF pathways [6]. Similarly, genomic studies of ovarian cancer also revealed frequent *TP53* mutations in high-grade serous ovarian carcinomas [9], and the prevalence of mutations affecting the PTEN/PI3K, WNT, RAS, and SWI/SNF pathways in non-serous ovarian cancers (e.g., endometrioid ovarian cancer) [10]. Of note, it was found that uterine serous carcinomas share many genomic features with ovarian serous carcinomas and basal-like breast cancers [6]; however, the biological basis for this similarity remains elusive. Lastly, mutations affecting the p53, PTEN/PI3K, and RAS pathways were also found in uterine and ovarian carcinosarcomas [7,8].

It has been long recognized that cancer cells often exhibit traits reminiscent of normal stem cells, such as self-renewal and the capacity for multi-lineage differentiation [11]. Cancer can initiate either from tissue stem cells, which already possess these properties, or from lineage-committed, more differentiated cells, after the acquisition of stem cell-like properties (i.e., “stemness”), leading to the formation of multipotent stem-like cancer cells (i.e., tumor-initiating cells (TICs) or cancer stem cells (CSCs)) [12]. TICs/CSCs are evolved from the cellular origin of cancer, and, conceptually, TICs/CSCs and the cells-of-origin of cancer are distinct [13]. TICs/CSCs are a subset of cancer cells that possess self-renewal capacities and can contribute to the heterogeneous lineages of cancer cells that comprise the tumor, whereas the cells-of-origin of cancer are normal cells that upon acquisition of oncogenic events (e.g., genetic mutations, aberrant epigenetic changes), eventually give rise to TICs/CSCs. Thus, in order to understand TICs/CSCs of a particular cancer type, it is essential to identify its cellular origin and to study the interplay of its cell-of-origin and cancer-associated driver mutations.

The cellular origins of gynecological malignancies are complicated and remain controversial. In ovarian cancers, initially, ovarian surface epithelial (OSE) cells and, more recently, fallopian tube epithelial (FTE) cells were proposed as cells-of-origin [14,15,16,17,18,19,20]. Both mesothelial cells (i.e., cells lining the body’s serous cavities and internal organs) and Müllerian duct-derived epithelial cells from the ovary, fallopian tube (FT), and uterus can potentially give rise to uterine and ovarian cancers. OSE cells and cells lining the FT serosa (i.e., mesothelial lining) are both mesothelial cells in nature. However, they are related developmentally to Müllerian-derived epithelial cells lining the lumens of the FT, uterus (endometrium), and uterine cervix, as both mesothelial and epithelial cells in the female reproductive system are developed from the coelomic epithelium of the early embryo [14]. The similarity between the mutational landscapes of uterine and ovarian cancers suggests that certain oncogenic mutations may play a dominant role in determining the subtype of gynecological cancer that may develop. To date, how driver mutations identified from sequencing studies and their corresponding deregulated pathways affect gynecological cancer development from their cellular origin remains largely elusive. In this study, we developed a genetic approach in mice to target both mesothelial and Müllerian-derived epithelial cells in the female reproductive system and asked how different oncogenic events affect the types of gynecological cancer that may develop.

## 2. Materials and Methods

### 2.1. Mouse Lines and Procedures

Wild-type FVB mice were purchased from Charles River Laboratories. *Rosa26-LSL-tdTomato* (*R26tdTm*) (JAX stock #: 007914), *K8-CreER^T2^* (JAX stock #: 017947), and *Lgr5-EGFP-IRES-CreER^T2^* (*Lgr5-GC*) (JAX stock #: 008875) mice were purchased from The Jackson Laboratory (JAX). Adenoviruses were introduced into their ovarian bursa via intrabursal injection. For CreER induction studies, a single dosage of 1 mg tamoxifen (T5648, Sigma, St. Louis, MO, USA) was administered to either *K8-CreER^T2^;R26tdTm* or *Lgr5-GC;R26tdTm* mice by intraperitoneal (IP) injection. For intrabursal injection, 5 × 10^7^ pfu *Ad-K8-Cre* or *Ad-CMV-Cre* was mixed with DMEM/F12 medium and Trypan blue (adenovirus injection mix) to visualize the performance of the surgical procedure and 5 μL of the adenovirus injection mix was injected into the intrabursal space of female mice. *Ad-CMV-Cre* and *Ad-K8-Cre* [21] adenoviruses were purchased from the Gene Transfer Core of the University of Iowa. *Trp53^loxP/loxP^* (JAX strain #: 008462), *Rb1^loxP/loxP^* (JAX strain #: 008186), *Brca1^loxP/loxP^* (JAX strain #: 017835), *Pten^loxP/loxP^* (JAX strain #: 004597), *Dicer^loxP/loxP^* (JAX strain #: 006366), *Apc^loxP/loxP^* (JAX strain #: 009045), Rosa26-LSL-^Kras*G12D/+^ (JAX strain #: 008179), and Rosa26-LSL-^Pik3ca*H1047R/+^(JAX strain #: 016977) mice were purchased from JAX. All experimental procedures reported herein were reviewed and approved by Brigham and Women’s Hospital institutional animal care and use committee (IACUC) and performed in accordance with the relevant protocol.

### 2.2. Ovarian Surface Epithelial (OSE) Cell Preparation

Whole ovaries were dissected from 8- to 10-week-old female mice and cell suspensions were prepared as previously described with minor modifications [22,23]. Briefly, whole ovaries were incubated with pre-warmed 0.25% trypsin/EDTA (Invitrogen, Carlsbad, CA, USA) at 37 °C and 5% CO_2_ for 30 min. Following trypsin inactivation with DMEM/F12 supplemented with 10% FBS, the supernatant containing stripped OSE cells was filtered through a 100 μm strainer, followed by a 40 μm strainer to achieve a single-cell suspension. Each cell suspension was pelleted at 1500 g for 5 min at 4 °C, and then analyzed by fluorescence-activated cell sorting (FACS).

### 2.3. Immunofluorescence and Immunohistochemical Analysis

Immunofluorescence analysis was performed as described previously [21]. Briefly, whole ovaries and fallopian tubes were fixed in 10% formalin and embedded in paraffin. Antibodies for K8 (904801, 1:1000, BioLegend, San Diego, CA, USA), YFP/GFP (Ab290, 1:200, Abcam, Cambridge, MA, USA), Tomato (Ab62341, 1:300, Abcam), p-AKT (D9E, 1:100, Cell signaling, Danvers, MA, USA), PAX8 (10336-1-AP, 1:1200, Proteintech, Rosemont, IL, USA), ERα (SC542, 1:50, Santa Cruz, Dallas, TX, USA), and β-catenin (610154, 1:200, BD bioscience, Billerica, MA, USA) were used. Antigen retrieval (Citrate buffer pH 6.0, 20 min boil in a microwave oven) was performed prior to incubation with the above-listed antibodies.

### 2.4. Fluorescence-Activated Cell Sorting

Flow cytometric analysis and FACS sorting were performed with a FACSAria sorter (BD Biosciences). Data were analyzed with FlowJo (Tree Star, Ashland, OR, USA). Antibodies used for FACS were purchased from eBiosciences (San Diego, CA, USA) and included biotinylated CD31 (13-0311-85), CD34 (13-0341-82), CD45 (13-0451-85), and TER119 (13-5921-85) (i.e., lineage [Lin] markers), as well as Streptavidin-PerCP-CY5.5 (45-4317-82).

### 2.5. Data Presentation and Analysis

All experiments were independently replicated at least three times. Data were reported as mean ± SEM. While qualitative images presented are representative of the outcomes obtained in the replicate experiments.

## 3. Results

### 3.1. Intrabursal Injection of Ad-K8-Cre Targets Epithelial and Mesothelial Cells in the Ovary, FT, and Uterus

**Figure 1:**
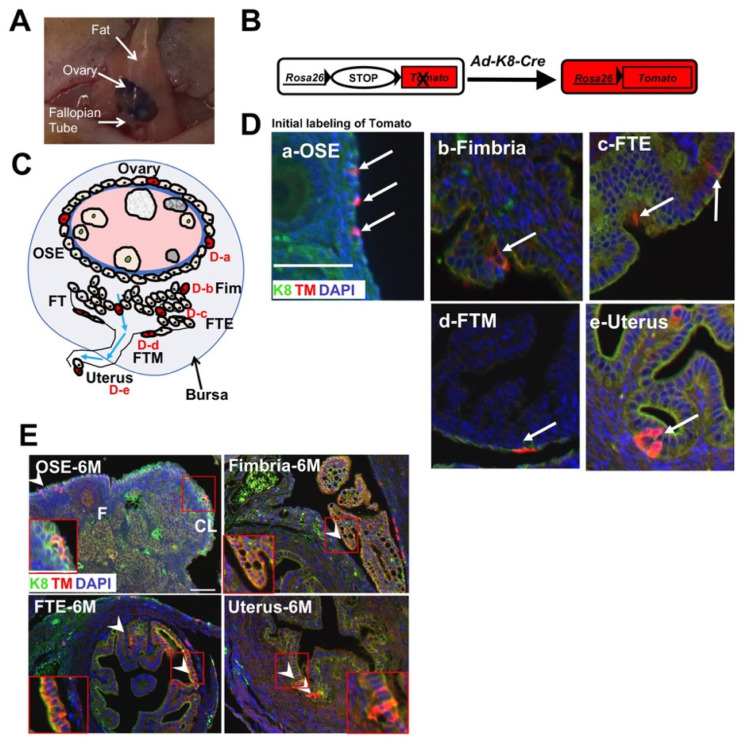
*Ad-K8-Cre* targets mesothelial and Müllerian-derived epithelial cells. (**A**) Mouse ovary and associated tissues are shown as an example of intrabursal injection (with Trypan blue). (**B**) Schematic diagram showing the activation of the Tomato reporter from the *R26tdTm* allele upon Cre expression from the *Ad-K8-Cre* virus. X for Tomato on the left right indicates that the Tomato reporter is initially silenced. (**C**) Graphical diagram showing expected cells infected by *Ad-K8-Cre* upon intrabursal injection. Staining data are shown in (**D**). OSE: ovarian surface epithelium; Fim: Fimbria; FTE: fallopian tube epithelium; FTM: Fallopian tube mesothelium. (**D**) Representative images of the ovary (**a**), fallopian tube (**b**–**d**), and uterus (**e**) from *R26tdTm* female mice showing K8, Tomato (TM) (arrows, indicating Tomato^+^ cells), and DAPI staining 2 weeks after *Ad-K8-Cre* intrabursal injection. F: follicle. Scale bar = 50 μm. (**E**) Representative images of the ovary, FT, and uterus from *R26tdTm* reporter mice six months (6 M) after intrabursal injection of *Ad-K8-Cre*, showing K8, Tomato (TM, as a lineage tracing marker), and DAPI staining. Arrowheads indicate *Ad-K8-Cre*-infected cells and/or their progeny (upon tracing, TM^+^). F: follicle; CL: corpus luteum. Scale bar (applies to all pictures in the same Figure panel) = 50 μm.

To model the development of ovarian or uterine cancer in mice, a commonly used approach is the intrabursal or intrauterine injection of Cre-expressing adenovirus under the control of the constitutive *CMV* promoter (*Ad-CMV-Cre*), respectively, to mice carrying floxed alleles of various tumor suppressors or oncogenes [24,25,26,27,28,29,30]. However, upon intrabursal injection (Figure 1A), in addition to ovarian cancers, malignancies in the uterus were reported as well [25,28]. In these model systems, as *Ad-CMV-Cre* can target epithelial cells, mesothelial cells, stromal cells, and other cell types, it is unclear whether the ovarian or uterine cancers observed in them were derived only from epithelial and/or mesothelial cells or from other cell types; second, upon intrabursal injection, it is unclear whether the observed uterine cancers were derived from endometrial cells or from other cell types, or due to metastatic spreading. To directly study the development of these gynecologic cancers from epithelial and mesothelial cells only, we utilized a new Cre-expressing adenovirus under the control of the *Krt8* promoter (*Ad-K8-Cre*) [21]. Recently, it was reported that the ALDH^+^ subpopulation of OSE cells contained OSE stem cells expressing LGR5 [22]. By analyzing the microarray expression profiling data from this study based on comparing ALDH^+^ to ALDH^−^ OSE cells, we found that *Krt8* is a keratin gene expressed at higher levels in ALDH^+^ OSE cells (see Supplementary Material, Figure S1A). We performed immunofluorescence (IF) staining for its gene product, Keratin 8 (K8), and confirmed its expression in the OSE cells of the ovary; in addition, we found that K8 is also expressed in the FT epithelial cells (in both the fimbria and tubal region of the FT) and serosa mesothelial lining cells, as well as in the endometrial glandular cells in the uterus (Figure S1B).

To determine the cell types targeted by *Ad-K8-Cre* upon intrabursal injection, we introduced *Ad-K8-Cre* to *Rosa26-LSL-tdTomato* (*R26tdTm*) reporter mice (Figure 1B). Upon intrabursal injection, adenoviruses would present in the space between the ovary/FT and the membranous bursa (as illustrated by the included blue dye, Figure 1A), and may also present in the reproductive tract through the opening of the FT within the intrabursal space (Figure 1C). Several days after injection, by the IF staining of the “lineage tracer” Tomato, we found that *Ad-K8-Cre* targeted OSE cells, as well as both fimbrial and tubal epithelial cells and tubal serosa mesothelial cells (of the FT) (Figure 1D(a–d)). We also observed Tomato-labeled endometrial epithelial cells in the uterus (Figure 1D(e)), suggesting the intrabursally injected *Ad-K8-Cre* could reach cells in the uterus for Cre-mediated recombination. Next, we performed pulse/chase lineage tracing of *Ad-K8-Cre*-targeted epithelial/mesothelial cells. Upon chasing for six months, we could still detect Tomato-marked OSE cells in the ovary, Tomato-marked fimbrial/tubal epithelial cells, and serosa mesothelial cells in the FT, as well as Tomato-marked endometrial epithelial cells, and the labeled cells exhibited signs of clonal expansion (i.e., clones of multiple Tomato-marked cells) (Figure 1E). In an independent approach, by the tamoxifen-induced activation of Cre-estrogen receptor fusion (CreER) under the control of the same *Krt8* promoter (in *K8-CreER^T2^;R26tdTm* mice), we also observed the genetic marking of OSE cells, FT epithelial, and serosa mesothelial cells, as well as endometrial cells both upon the initial labeling and after chasing for several months (Figure S2A,B). Together, these data suggest that *Ad-K8-Cre* could target both mesothelial cells and Müllerian-derived epithelial cells in the ovary, FT, and uterus.

### 3.2. Intrabursal Injection of Ad-K8-Cre Preferentially Targets LGR5^+^ Stem Cells in the Ovary

**Figure 2:**
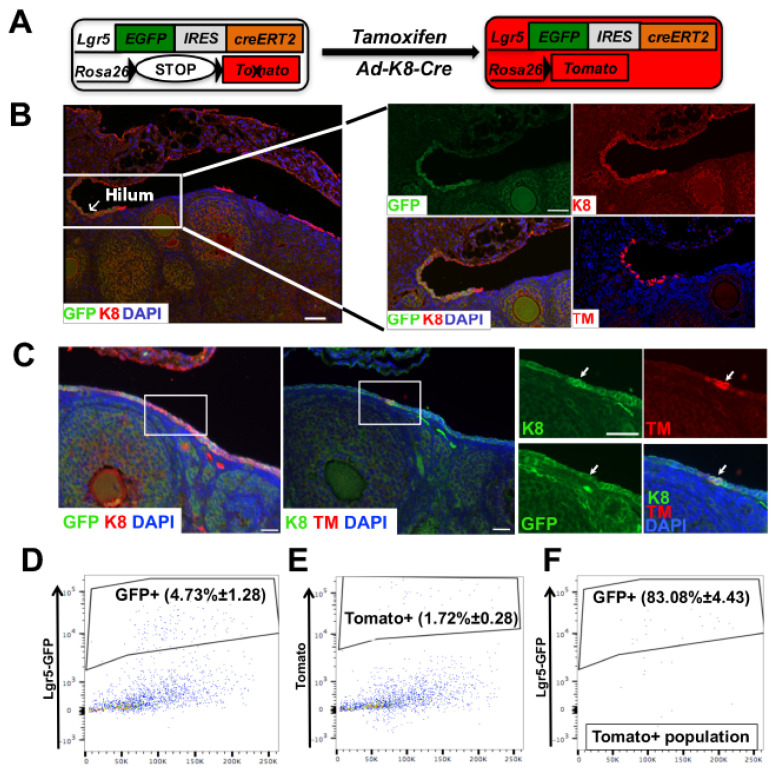
*Ad-K8-Cre* preferentially targets LGR5^+^ OSE cells. (**A**) Schematic diagram showing the activation of the Tomato reporter in the *Lgr5-GC;R26tdTm* model, either upon tamoxifen-induced activation of CreER in *Lgr5-GC* or upon Cre expression from the injected *Ad-K8-Cre* virus. (**B**,**C**) Representative images of the ovarian hilium region (**B**) and OSE region (**C**) from *Lgr5-GC;R26tdTm* mice showing K8, GFP (*Lgr5*), TM (Tomato) (*Ad-K8-Cre*-infected cells), and DAPI staining 2 weeks after *Ad-K8-Cre* intrabursal injection. Arrows indicate GFP (*Lgr5*) and K8 double-positive cells. Scale bars = 50 μm. (**D**–**F**) FACS analysis of OSE cells from *Lgr5-GC;R26tdTm* mice 4 days after *Ad-K8-Cre* injection showing GFP (*Lgr5*)^+^ cells (**D**), Tomato^+^ cells (**E**), and GFP^+^Tomato^+^ cells (**F**).

Ovarian cancer may originate from either OSE cells or FTE cells [14,15,16,17,18,19,20]. In the OSE lineage, a subpopulation of LGR5^+^ cells have been identified as the OSE stem cells to sustain this lineage and have also been implicated as the cellular origin of ovarian cancer (particularly those in the ovarian hilum region) [22,23]. To determine whether we can target LGR5^+^ cells in the ovary by using *Ad-K8-Cre*, we employed the *Lgr5-eGFP-CreER^T2^;R26tdTm* (*Lgr5-GC;R26tdTm*) mouse model. In this model, the Tomato reporter can be turned on by the Cre activity from either tamoxifen induction (i.e., to label LGR5^+^ progenitor cells by CreER activation) or the intrabursal injection of *Ad-K8-Cre* (i.e., to label *Ad-K8-Cre*-infected K8^+^ cells by Cre expression from *Ad-K8-Cre*) (Figure 2A). For the latter, the *Lgr5-GC* allele only serves as a reporter to indicate *Lgr5*^+^ cells (as CreER from this allele is not activated without tamoxifen), and upon *Ad-K8-Cre* injection, we can visualize endogenous *Lgr5* expression (shown as GFP) and K8^+^ cells infected *by Ad-K8-Cre* (shown as Tomato^+^) simultaneously (Figure 2A).

To determine the base levels of K8 and *Lgr5*(GFP) expression in *Lgr5-GC;R26tdTm* female mice, we performed co-IF staining and found that K8 and *Lgr5*(GFP) were co-expressed in a subset of cells at the OSE and hilum region (Figure 2B,C). Next, we performed a short-term lineage tracing (2 weeks) in *Lgr5-GC;R26tdTm* mice via a single-dose injection of tamoxifen [22,23]. By the co-IF staining of K8, GFP, and Tomato, we observed that in the hilum region, *Lgr5*(GFP)^+^ cells, labeled by Tomato, were also positive for K8 (Figure 2B); in the OSE region, we also observed that some *Lgr5*(GFP)^+^K8^+^ cells became positive for Tomato (Figure 2C, right). Lastly, we performed the intrabursal injection of *Ad-K8-Cre* to *Lgr5-GC;R26tdTm* female mice. To quantify how many *Ad-K8-Cre*-targeted OSE cells were *Lgr5*(GFP)^+^ cells, we performed FACS analysis for the injected females and found that the majority of *Ad-K8-Cre*-infected cells (Tomato^+^ cells) were *Lgr5*(GFP)^+^ cells (83.08 ± 4.43%, *n* = 4) (Figure 2D–F). This observation is thus consistent with the previous expression profiling study showing higher *Krt8* expression in ALDH^+^ OSE cells, which are enriched with LGR5^+^ OSE stem cells (Figure S1A) [22]. Together, these data suggest that upon the intrabursal injection of *Ad-K8-Cre*, the majority of the infected K8^+^ cells in the ovary are also LGR5^+^ cells (possibly due to the higher activity of *Krt8* promoter in these cells).

### 3.3. Summary of Ovary/FT/Uterus Histology in Various Mouse Models upon the Intrabursal Injection of Ad-K8-Cre

**Figure 3:**
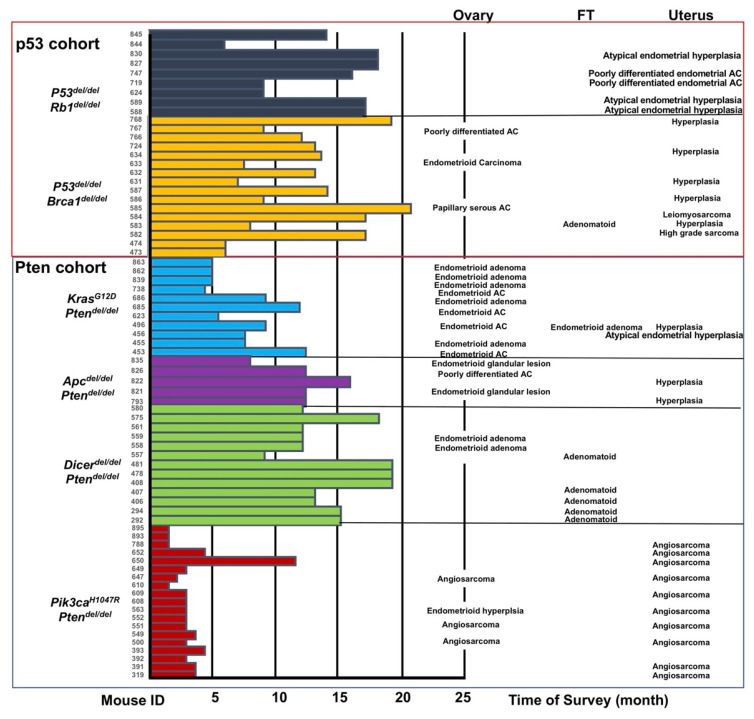
Histological summary of gynecological malignancies induced by the intrabursal injection of *Ad-K8-Cre* to various mouse models. The histogram indicates the time of the survey of individual mice. Lesions and phenotypes are shown in the corresponding tissues. The same color indicates the same mouse model. FT: fallopian tube; AC: adenocarcinoma; del: deletion.

To model ovarian cancer development, several mouse models based on the intrabursal injection of *Ad-CMV-Cre* were previously generated. For instance, *Ad-CMV-Cre*-mediated inactivation of *Trp53* and *Rb1* in OSE cells led to the development of ovarian tumors resembling human serous adenocarcinoma [24]. The *Ad-CMV-Cre*-mediated inactivation of *Pten* and the activation of *Kras^G12D^* in OSE cells, or the inactivation of both *Pten* and *Apc*, led to the development of endometrioid ovarian cancers [25,26]. Furthermore, the *Ad-CMV-Cre*-mediated inactivation of *Pten* and the activation of *Pik3ca^H1047R^* led to the development of ovarian serous adenocarcinomas or granulosa cell tumors [27]. Lastly, the *Ad-CMV-Cre*-mediated inactivation of both *Brca1* and *p53* only led to the development of ovarian leiomyosarcomas [28,31]; however, when the *Ad-CMV-Cre*-mediated inactivation of *p53* and *Brca1* was combined with the inactivation of Rb1 and p107/p130 pocket proteins, the animals developed advanced serous ovarian cancers [29]. As *Ad-CMV-Cre* can target both epithelial and non-epithelial cells and, as we show, *Ad-K8-Cre* is more specific for epithelial/mesothelial cells in the ovary (mainly LGR5^+^ OSE stem cells), FT, and uterus (Figure 1 and Figure 2), we performed the intrabursal injection of *Ad-K8-Cre* into engineered mice carrying the following combinations of oncogenic events: *Trp53^loxP/loxP^;Rb1^loxP/loxP^* (p53/Rb1), *Trp53^loxP/loxP^;Brca1^loxP/loxP^* (p53/Brca1), Rosa26-LSL-^Kras*G12D/+^(*Kras^G12D/+^*)*;Pten^loxP/loxP^* (Pten/Kras), *Apc^loxP/loxP^;Pten^loxP/loxP^* (Pten/Apc), Rosa26-LSL-^Pik3ca*H1047R/+^ (*Pik3ca^H1047R/+^*)*;Pten^loxP/loxP^* (Pten/Pik3ca), and *Dicer^loxP/loxP^;Pten^loxP/loxP^* (Pten/Dicer). We monitored the injected females for up to 18 months; some mice were euthanized earlier for analysis due to various health issues, including tumor development. Upon termination, we collected their ovaries, FTs, and uteri for histological analyses. In most injected females, we found at least one malignancy in either their uteri or ovaries and, to a lesser degree, in their FTs (summarized in Figure 3).

### 3.4. Gynecological Malignancies Developed in the p53 Cohort

**Figure 4:**
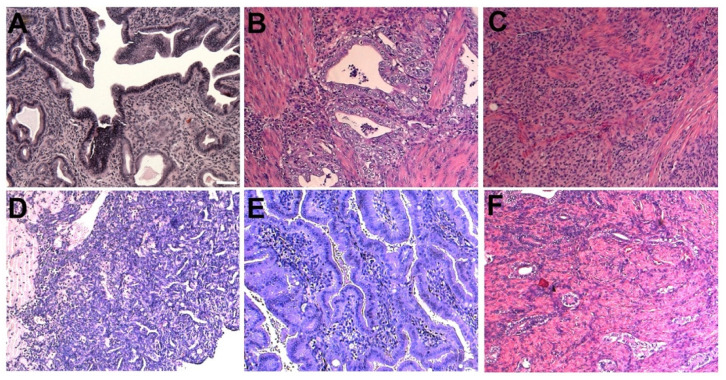
Gynecological malignancies developed in the p53 cohort after *Ad-K8-Cre* injection. (**A**–**C**) Representative histological images of hyperplasia (**A**), endometrial adenocarcinoma (**B**), and leiomyosarcoma (**C**) in the uterus. (**D**,**E**) Representative histological images of endometrioid carcinoma (**D**) and serous adenocarcinoma in the ovary (**E**) from the injected p53/Brca1 mice. (**F**) Representative histological image of atypical endometrial hyperplasia in the uterus from the injected p53/Rb1 females. Scale bar (same for all panels) = 50 μm.

Our mouse models can be divided into two cohorts: one cohort was based on the Cre-induced loss of *Trp53* (i.e., p53 cohort) coupled with the loss of either *Rb1* or *Brca1*, the other was based on the Cre-mediated loss of *Pten* (i.e., Pten cohort) coupled with several other oncogenic events (i.e., the loss of *Apc* or *Dicer*, or the activation of *Pik3ca^H1047R^* or *Kras^G12D^* mutation) (Figure 3). In the p53 cohort, we found that most gynecological malignancies were observed in the uterus (Figure 3, upper). In the injected p53/Brca1 mice, we found that ~half of them developed various endometrial malignancies, including the hyperplasia of endometrial cells (Figure 4A), endometrial adenocarcinoma (Figure 4B), and leiomyosarcoma (Figure 4C). A small number of the injected p53/Brca1 mice developed ovarian cancers, including poorly differentiated adenocarcinoma, endometrioid (Figure 4D), and serous adenocarcinomas (Figure 4E). Immunostaining confirmed K8 and nuclear PAX8 expression in the serous tumor (Figure S3A,B). In this experimental system, we could not determine exactly whether these ovarian malignancies were derived from *Ad-K8-Cre*-infected OSE cells or *Ad-K8-Cre*-infected FT cells. However, multiple lines of evidence from previous studies have rendered support to a tubal origin of these lesions (e.g., based on *Pax8* or *Ovgp1*-based genetic targeting), in particular the serous adenocarcinoma [16,17,18,19]. Thus, it is possible that most of the ovarian cancers observed in the injected p53/Brca1 mice originated from Müllerian-derived epithelial cells (i.e., FTE cells). In the injected p53/Rb1 mice, we only detected malignancies in their uteri, including atypical endometrial hyperplasia (Figure 4F) and poorly differentiated adenocarcinoma. Immunostaining confirmed K8 and ERα expression in the uterine endometrioid cancer (Figure S3C,D). We did not observe serous cancer in the ovaries of our injected p53/Rb1 mice, possibly due to the use of a different conditional *Rb1* knockout model from that used in a previous study (which developed serous ovarian cancer) [24]. Due to the anatomy, the intrabursally injected *Ad-K8-Cre* would mainly target mesothelial cells (including OSE cells and FT serosa lining cells), followed by FTE cells in the fimbria (which is exposed in the intrabursal space), and then FTE cells in the tubal region, as well as endometrial cells in the uterus (Figure 1C), yet the majority of malignancies detected in the p53 cohort were found in the uterus (as well as in the ovary, whose lesions may have an FTE origin). These observations raise the possibility that Müllerian-derived epithelial cells, such as endometrial cells (and possibly also FTE cells, which give rise to “ovarian” cancer), may be particularly sensitive to the loss of p53.

### 3.5. Gynecological Malignancies Developed in the Pten Cohort

**Figure 5:**
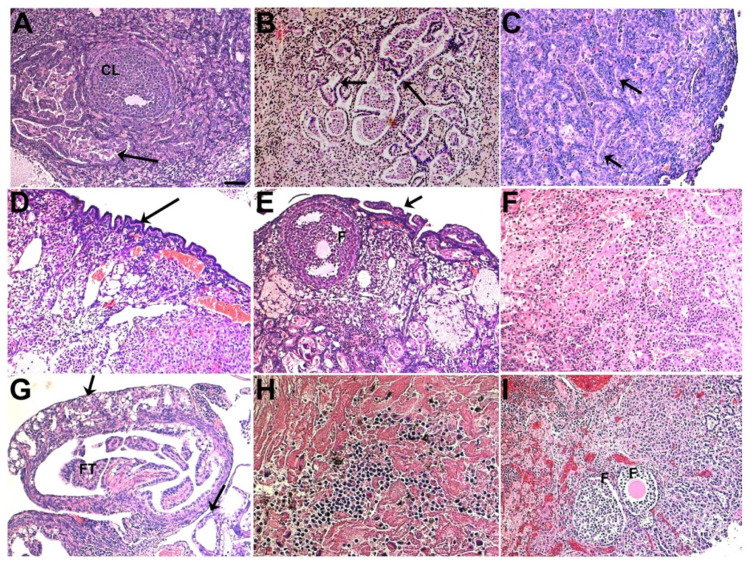
Gynecological malignancies developed in the Pten cohort after *Ad-K8-Cre* injection. (**A**–**C**) Representative histological images of endometrioid adenocarcinomas in the ovary from the injected Pten/Kras mice. Arrows indicate villoglandular structures. (**D**–**F**) Endometrioid hyperplasia ((**D**), arrow), endometrioid finger-like lesion ((**E**), arrow), and poorly differentiated adenocarcinoma (**F**) in the ovary from the injected Pten/Apc mice. (**G**) Adenomas (i.e., adenomatoid-like lesions) developed on the surface of the FT ((**G**), arrows) from the injected Pten/Dicer mice. (**H**,**I**) Angiosarcoma in the uterus (**H**) and angiosarcoma/hemorrhage in the ovary (**I**) from the injected Pten/Pik3ca mice. F: follicle; CL: Corpus luteum; FT: fallopian tube. Scale bar (same for all panels) = 50 μm.

In contrast, in the Pten cohort, we observed gynecological malignancies that are potentially more related to the transformation of mesothelial cells (Figure 3, lower). In both the Pten/Kras and Pten/Apc models, most malignancies were observed in their ovaries, including endometrioid hyperplasia, adenoma, and adenocarcinoma (negative for PAX8 staining), as well as poorly differentiated adenocarcinoma (Figure 5A–C: Pten/Kras; Figure 5D–F, Pten/Apc). We detected very few lesions in their FTs and uteri (for the latter, with the exception of the Pten/Pik3ca model, see below). In the Pten/Apc model, it was shown previously that both OSE cells (based on the intrabursal injection of *Ad-CMV-Cre*) and FTE cells (based on the tamoxifen-induced activation of *Ovgp1-iCreER*) could serve as the cells-of-origin of endometrioid ovarian cancer [26,32]. In this study, as the intrabursal injection of *Ad-K8-Cre* mainly targeted OSE cells (thus more comparable to the previous *Ad-CMV-Cre* intrabursal injection-based study)—in particular, LGR5^+^ OSE stem cells (Figure 2)—our observation raises the possibility that LGR5^+^ OSE stem cells, which are mesothelial cells in nature, may be particularly sensitive to loss of Pten, leading to their transdifferentiation toward endometrioid-like cells.

In the Pten/Dicer model, we also observed endometrioid-like lesions in the ovaries of a small number of the injected mice (Figure 3 and Figure S4A,B, endometrioid hyperplasia and adenoma). Interestingly, ~half of the injected Pten/Dicer mice developed adenoma on the surface of their FTs, and these malignancies appeared to originate from tubal serosa mesothelial cells (Figure 5G). These adenomas resemble adenomatoid tumors found in human patients, which are the most common type of benign tumor of the FT in women [33,34]. The genetics of human FT adenomatoid tumors is unclear and our study may provide a genetic basis for this type of FT malignancy (e.g., the loss of Dicer (or disruption of the microRNA processing machinery, or loss of certain key mature microRNAs as a result) plus the loss of Pten). Previously, it was reported that transgenic mice carrying *Amhr2-Cre* that induced the deletion of *Dicer^loxP/loxP^* and *Pten^loxP/loxP^* alleles in FT stromal cells developed serous ovarian adenocarcinomas [35]. Since *Amhr2* starts its expression in the mesenchyme of Müllerian ducts during the embryonic stage and later in the granulosa cells of the ovary and smooth muscle and stromal cells in the FT postnatally [36,37,38,39], the deletion of *Dicer* and *Pten* in various cell types during their embryonic life from this previous study could be different from ours, in which mice were induced for mutagenesis specifically in mesothelial cells and Müllerian-derived epithelial cells during their adult life [35].

In the Pten cohort, the injected mice that developed the quickest clinical signs were those injected Pten/Pik3ca females. Within two months post-injection, most injected Pten/Pik3ca females were in drastically bad health condition with bloody messes including bloody ovaries in the peritoneum and were either found dead or sacrificed for analysis. Histological analysis found that the majority of them developed angiosarcomas in their uteri (Figure 3 and Figure 5H). In the ovaries of a small number of these mice, we also observed endometrioid hyperplasia, in addition to extensive hemorrhage and angiosarcoma (Figure 5I). Of note, it was reported previously that in a similar mouse model, hemorrhagic ascites with intraperitoneal masses were also observed [27]. The exact nature of the angiosarcoma and ovarian hemorrhage is unknown.

Lastly, in the Pten cohort, by immunohistochemical staining, we confirmed the expression of K8 and phospho-AKT (p-AKT, due to Pten-loss) in the ovarian malignancies in the injected Pten/Kras mice (Figure S4C,D); the expression of K8, p-AKT, and nuclear β-catenin (due to Apc-loss) in the ovarian malignancies in the injected Pten/Apc mice (Figure S4E–G); and the expression of K8 and p-AKT in the ovarian malignancies in the injected Pten/Dicer mice (Figure S4H,I), as well as in ovaries of the injected Pten/Pik3ca mice (Figure S4J,K).

## 4. Discussion

In this study, we performed a comparative study of gynecological malignancies developed in the ovaries, FTs, and uteri of the *Ad-K8-Cre*-injected mice, by recapitulating mutational events commonly found in human uterine and ovarian cancers. Our study is unique, as the genetic approach we used specifically targets the K8^+^ epithelial and mesothelial cells in the ovary, FT, and uterus. Our lineage-tracing experiments showed that the intrabursally injected *Ad-K8-Cre* not only targeted LGR5^+^ OSE stem cells but might also target long-lived stem cells in the FT epithelial and endometrial epithelial lineages, which are potential targets of transformation for their corresponding cancer types. The malignant phenotypes observed in various models possibly represent the unique interplay of different oncogenic events and various K8^+^ cell types in the ovary, FT, and uterus.

Collectively, we can draw several conclusions. First, oncogenic events—in particular, dominant acting oncoproteins (e.g., mutant KRAS, mutant PIK3CA)—often dictate the malignant phenotypes in the ovary/reproductive tract. The cancer latencies driven by oncoproteins are much shorter than those from models induced by the inactivation of only tumor suppressors (Figure 3). In most injected Pten/Kras mice, we detected endometrioid-like lesions in their ovaries after a relatively short latency. These lesions could originate from OSE cells (particularly LGR5^+^ OSE cells) or FTE cells. Regardless of the cellular origin, the predominant cancer phenotype seems to be the endometrioid-like malignancy found in the ovary, suggesting that the activating KRAS mutant protein may dominantly dictate the malignant phenotype. Although we observed almost no malignancies in the uteri of these mice, we cannot rule out the possibility that such mice may also develop uterine malignancies, but after a longer latency (than that of ovarian malignancies). In almost all injected Pten/Pik3ca females, we observed aggressive angiosarcomas in their uteri after a very short latency; we also detected some angiosarcomas in the ovaries of the injected mice, possibly as a result of the metastatic spreading of angiosarcoma cells from the uterus to the ovary. This is the only exception in the Pten cohort in which the predominant malignancy was observed in the uterus (Figure 3). Since other combinations in the Pten cohort did not exhibit this phenotype, the observed angiosarcoma might be largely induced by the PIK3CA^H1047R^ mutant oncoprotein (either alone or in combination with Pten-loss), which may largely affect K8^+^ endometrial cells. Although we did not observe genetically marked endothelial cells in the uterus upon the intrabursal injection of *Ad-K8-Cre*, we cannot entirely rule out the possibility that the observed angiosarcoma may be derived from very rare endothelial cells targeted by *Ad-K8-Cre*.

Second, in almost all injected models in the Pten cohort, we observed endometrioid-like lesions in their ovaries, including low-grade endometrioid hyperplasia and adenoma. This trend became more profound when Pten-loss was combined with either the activation of the KRAS mutant or the loss of APC (Figure 3). Even in the injected Pten/Pik3ca mice, we observed at least one case of ovarian endometrioid hyperplasia (together with angiosarcoma detected in its uterus, Figure 3). These mice might have developed more endometrioid-like lesions in their ovaries if they were not euthanized earlier due to their aggressive angiosarcoma lesions. Clinicopathologically, a dualistic model of ovarian cancer has been proposed: Type I tumors, comprised of low-grade serous, low-grade endometrioid, mucinous, and clear cell carcinomas, typically possess mutations in *KRAS*, *PTEN*, and *CTNNB1* [40]. Our data are consistent with this model of low-grade ovarian cancer. Of note, although these ovarian endometrioid-like lesions may originate from either OSE cells or FTE cells, they are more likely to come from the transformation of OSE cells, as intrabursally injected *Ad-K8-Cre* would mainly target OSE cells—in particular, LGR5^+^ OSE cells (Figure 2). Nevertheless, similar to a dominant acting oncoprotein, Pten-loss here also appears to be “dominating” in preferentially inducing an endometrioid-like malignant phenotype in the ovary.

Lastly, it appears that Müllerian-derived K8^+^ FTE cells and K8^+^ endometrial cells, in particular the latter, are more sensitive to the loss of p53 (than mesothelial cells). The loss of p53 in K8^+^ endometrial cells leads to the development of various endometrial malignancies, possibly including poorly differentiated adenocarcinoma and sarcoma, whereas the loss of p53 in K8^+^ FTE cells may lead to the development of serous adenocarcinoma and endometrioid adenocarcinoma in the ovary. In contrast, Pten-loss may affect mesothelial cells (e.g., OSE cells, mesothelial cells lining the FT) more than Müllerian-derived epithelial cells. Recently, by culturing FTE and OSE cells as organoids and by introducing the same sets of mutations (i.e., p53 mutation, either alone or in combination with other oncogenic events), it was found that both cell types could serve as the cells-of-origin of high-grade serous ovarian carcinomas (HGSOCs); however, mutated FTE cells exhibited a shorter latency and higher penetrance of developing HGSOCs than similarly mutated OSE cells [41,42]. Since p53 mutation was introduced to both FTE and OSE organoid cells, these observations are consistent with our finding that Müllerian-derived epithelial cells (e.g., FTE cells) may be particularly sensitive to the loss of p53 (compared to OSE cells, which are mesothelial cells in nature).

The preferential development of gynecological malignancies in a particular organ in response to specific oncogenic mutations may be caused by both cell-intrinsic (e.g., unique properties of the cell-of-origin) and extrinsic (e.g., microenvironment) mechanisms. For instance, the loss of both p53 and BRCA1 can lead to DNA damage and replication stress [43,44]; Müllerian-derived epithelial cells may be more sensitive to these challenges than mesothelial cells and, as a result, they are more likely to be transformed and become tumor-initiating cells in response to p53/BRCA1-loss. In addition, the accumulation of DNA damage in these epithelial cells can also induce immune reactions in the microenvironment (e.g., chronic inflammation), which can facilitate cancer initiation and progression in the affected organ. Of note, one caveat of our study is that we induced the total loss of p53 in our mouse models. In gynecological malignancies (e.g., HGSOCs), *TP53* mutations can be either loss-of-function or gain-of-function, and individual *TP53* hotspot mutations have different impacts on HGSOC patient outcomes [45]. Thus, future studies are needed to further elucidate whether *TP53* gain-of-function mutations would affect Müllerian-derived epithelial cells versus mesothelial cells in a similar or different way (when compared to the p53-loss from this work).

## 5. Conclusions

Overall, our study provides comprehensive insights into how common oncogenic events determine both the types and locations of gynecological malignancies via their unique interactions with cellular origins and supports a model that oncogenic events largely dictate the types and locations of gynecological malignancies.

## Data Availability

The publicly available microarray expression profiling data re-analyzed and shown in Figure S1A can be found from the GEO database website (https://www.ncbi.nlm.nih.gov/geo/) under the GEO accession number GSE43897.

## Supplementary Materials

The following are available online at https://www.mdpi.com/article/10.3390/cancers14030841/s1, Figure S1: K8 expression in mesothelial cells and Müllerian epithelial cells; Figure S2: Tracing of the *Krt8*^+^ lineage; Figure S3: Immunohistochemical analyses of marker expression in gynecological malignancies developed in the p53 cohort; Figure S4: Histological and immunohistochemical analyses of gynecological malignancies developed in the Pten cohort.

## References

[1] Timmermans M., Sonke G.S., Van de Vijver K.K., van der Aa M.A., Kruitwagen R. (2018). No improvement in long-term survival for epithelial ovarian cancer patients: A population-based study between 1989 and 2014 in the Netherlands. Eur. J. Cancer.

[2] Brucks J.A. (1992). Ovarian cancer. The most lethal gynecologic malignancy. Nurs. Clin. North Am..

[3] Siegel R., Naishadham D., Jemal A. (2012). Cancer statistics, 2012. CA Cancer J. Clin..

[4] Vaughan S., Coward J.I., Bast R.C., Berchuck A., Berek J.S., Brenton J.D., Coukos G., Crum C.C., Drapkin R., Etemadmoghadam D. (2011). Rethinking ovarian cancer: Recommendations for improving outcomes. Nat. Rev. Cancer.

[5] Erickson B.K., Conner M.G., Landen C.N. (2013). The role of the fallopian tube in the origin of ovarian cancer. Am. J. Obstet. Gynecol..

[6] Cancer Genome Atlas Research N., Kandoth C., Schultz N., Cherniack A.D., Akbani R., Liu Y., Shen H., Robertson A.G., Pashtan I., Shen R. (2013). Integrated genomic characterization of endometrial carcinoma. Nature.

[7] Cherniack A.D., Shen H., Walter V., Stewart C., Murray B.A., Bowlby R., Hu X., Ling S., Soslow R.A., Broaddus R.R. (2017). Integrated Molecular Characterization of Uterine Carcinosarcoma. Cancer Cell.

[8] Zhao S., Bellone S., Lopez S., Thakral D., Schwab C., English D.P., Black J., Cocco E., Choi J., Zammataro L. (2016). Mutational landscape of uterine and ovarian carcinosarcomas implicates histone genes in epithelial-mesenchymal transition. Proc. Natl. Acad. Sci. USA.

[9] Network C.G.A.R. (2011). Integrated genomic analyses of ovarian carcinoma. Nature.

[10] Teer J.K., Yoder S., Gjyshi A., Nicosia S.V., Zhang C., Monteiro A.N.A. (2017). Mutational heterogeneity in non-serous ovarian cancers. Sci. Rep..

[11] Shackleton M. (2010). Normal stem cells and cancer stem cells: Similar and different. Semin. Cancer Biol..

[12] Friedmann-Morvinski D., Verma I.M. (2014). Dedifferentiation and reprogramming: Origins of cancer stem cells. EMBO Rep..

[13] Visvader J.E. (2011). Cells of origin in cancer. Nature.

[14] Auersperg N. (2011). The origin of ovarian carcinomas: A unifying hypothesis. Int. J. Gynecol Pathol..

[15] Chene G., Dauplat J., Radosevic-Robin N., Cayre A., Penault-Llorca F. (2013). Tu-be or not tu-be: That is the question… about serous ovarian carcinogenesis. Crit. Rev. Oncol. Hematol..

[16] Perets R., Drapkin R. (2016). It’s Totally Tubular…Riding The New Wave of Ovarian Cancer Research. Cancer Res..

[17] Perets R., Wyant G.A., Muto K.W., Bijron J.G., Poole B.B., Chin K.T., Chen J.Y., Ohman A.W., Stepule C.D., Kwak S. (2013). Transformation of the fallopian tube secretory epithelium leads to high-grade serous ovarian cancer in Brca; Tp53; Pten models. Cancer Cell.

[18] Sherman-Baust C.A., Kuhn E., Valle B.L., Shih Ie M., Kurman R.J., Wang T.L., Amano T., Ko M.S., Miyoshi I., Araki Y. (2014). A genetically engineered ovarian cancer mouse model based on fallopian tube transformation mimics human high-grade serous carcinoma development. J. Pathol..

[19] Zhai Y., Wu R., Kuick R., Sessine M.S., Schulman S., Green M., Fearon E.R., Cho K.R. (2017). High-grade serous carcinomas arise in the mouse oviduct via defects linked to the human disease. J. Pathol..

[20] Karnezis A.N., Cho K.R., Gilks C.B., Pearce C.L., Huntsman D.G. (2017). The disparate origins of ovarian cancers: Pathogenesis and prevention strategies. Nat. Rev. Cancer.

[21] Tao L., van Bragt M.P.A., Laudadio E., Li Z. (2014). Lineage Tracing of Mammary Epithelial Cells Using Cell-Type-Specific Cre-Expressing Adenoviruses. Stem Cell Rep..

[22] Flesken-Nikitin A., Hwang C.I., Cheng C.Y., Michurina T.V., Enikolopov G., Nikitin A.Y. (2013). Ovarian surface epithelium at the junction area contains a cancer-prone stem cell niche. Nature.

[23] Ng A., Tan S., Singh G., Rizk P., Swathi Y., Tan T.Z., Huang R.Y., Leushacke M., Barker N. (2014). Lgr5 marks stem/progenitor cells in ovary and tubal epithelia. Nat. Cell Biol..

[24] Flesken-Nikitin A., Choi K.C., Eng J.P., Shmidt E.N., Nikitin A.Y. (2003). Induction of carcinogenesis by concurrent inactivation of p53 and Rb1 in the mouse ovarian surface epithelium. Cancer Res..

[25] Dinulescu D.M., Ince T.A., Quade B.J., Shafer S.A., Crowley D., Jacks T. (2005). Role of K-ras and Pten in the development of mouse models of endometriosis and endometrioid ovarian cancer. Nat. Med..

[26] Wu R., Hendrix-Lucas N., Kuick R., Zhai Y., Schwartz D.R., Akyol A., Hanash S., Misek D.E., Katabuchi H., Williams B.O. (2007). Mouse model of human ovarian endometrioid adenocarcinoma based on somatic defects in the Wnt/beta-catenin and PI3K/Pten signaling pathways. Cancer Cell.

[27] Kinross K.M., Montgomery K.G., Kleinschmidt M., Waring P., Ivetac I., Tikoo A., Saad M., Hare L., Roh V., Mantamadiotis T. (2012). An activating Pik3ca mutation coupled with Pten loss is sufficient to initiate ovarian tumorigenesis in mice. J. Clin. Invest..

[28] Quinn B.A., Brake T., Hua X., Baxter-Jones K., Litwin S., Ellenson L.H., Connolly D.C. (2009). Induction of ovarian leiomyosarcomas in mice by conditional inactivation of Brca1 and p53. PLoS ONE.

[29] Szabova L., Yin C., Bupp S., Guerin T.M., Schlomer J.J., Householder D.B., Baran M.L., Yi M., Song Y., Sun W. (2012). Perturbation of Rb, p53, and Brca1 or Brca2 cooperate in inducing metastatic serous epithelial ovarian cancer. Cancer Res..

[30] Cheng H., Liu P., Zhang F., Xu E., Symonds L., Ohlson C.E., Bronson R.T., Maira S.M., Di Tomaso E., Li J. (2014). A genetic mouse model of invasive endometrial cancer driven by concurrent loss of Pten and Lkb1 Is highly responsive to mTOR inhibition. Cancer Res..

[31] Clark-Knowles K.V., Senterman M.K., Collins O., Vanderhyden B.C. (2009). Conditional inactivation of Brca1, p53 and Rb in mouse ovaries results in the development of leiomyosarcomas. PLoS ONE.

[32] Wu R., Zhai Y., Kuick R., Karnezis A.N., Garcia P., Naseem A., Hu T.C., Fearon E.R., Cho K.R. (2016). Impact of oviductal versus ovarian epithelial cell of origin on ovarian endometrioid carcinoma phenotype in the mouse. J. Pathol..

[33] Ragins A.B., Crane R.D. (1948). Adenomatoid tumors of the fallopian tube. Am. J. Pathol..

[34] Rutgers J.K., Lawrence W.D. (2014). A small organ takes center stage: Selected topics in fallopian tube pathology. Int. J. Gynecol. Pathol..

[35] Kim J., Coffey D.M., Creighton C.J., Yu Z., Hawkins S.M., Matzuk M.M. (2012). High-grade serous ovarian cancer arises from fallopian tube in a mouse model. Proc. Natl. Acad. Sci. USA.

[36] Jamin S.P., Arango N.A., Mishina Y., Hanks M.C., Behringer R.R. (2002). Requirement of Bmpr1a for Mullerian duct regression during male sexual development. Nat. Genet..

[37] Jeyasuria P., Ikeda Y., Jamin S.P., Zhao L., De Rooij D.G., Themmen A.P., Behringer R.R., Parker K.L. (2004). Cell-specific knockout of steroidogenic factor 1 reveals its essential roles in gonadal function. Mol. Endocrinol..

[38] Deutscher E., Hung-Chang Y.H. (2007). Essential roles of mesenchyme-derived beta-catenin in mouse Mullerian duct morphogenesis. Dev. Biol..

[39] Arango N.A., Kobayashi A., Wang Y., Jamin S.P., Lee H.H., Orvis G.D., Behringer R.R. (2008). A mesenchymal perspective of Mullerian duct differentiation and regression in Amhr2-lacZ mice. Mol. Reprod. Dev..

[40] Kurman R.J., Shih I.M. (2016). The Dualistic Model of Ovarian Carcinogenesis: Revisited, Revised, and Expanded. Am. J. Pathol..

[41] Lohmussaar K., Kopper O., Korving J., Begthel H., Vreuls C.P.H., van Es J.H., Clevers H. (2020). Assessing the origin of high-grade serous ovarian cancer using CRISPR-modification of mouse organoids. Nat. Commun..

[42] Zhang S., Dolgalev I., Zhang T., Ran H., Levine D.A., Neel B.G. (2019). Both fallopian tube and ovarian surface epithelium are cells-of-origin for high-grade serous ovarian carcinoma. Nat. Commun..

[43] He Y., Rivera J., Diossy M., Duan H., Bowman-Colin C., Reed R., Jennings R., Novak J., Tran S.V., Cohen E.F. (2021). BRCA1/Trp53 heterozygosity and replication stress drive esophageal cancer development in a mouse model. Proc. Natl. Acad. Sci. USA.

[44] Wang H., Xiang D., Liu B., He A., Randle H.J., Zhang K.X., Dongre A., Sachs N., Clark A.P., Tao L. (2019). Inadequate DNA Damage Repair Promotes Mammary Transdifferentiation, Leading to BRCA1 Breast Cancer. Cell.

[45] Tuna M., Ju Z., Yoshihara K., Amos C.I., Tanyi J.L., Mills G.B. (2020). Clinical relevance of TP53 hotspot mutations in high-grade serous ovarian cancers. Br. J. Cancer.

