# Supplementary material for: Oncogenic Events Dictate the Types and Locations of Gynecological Malignancies Originating from Krt8+ Mesothelial and Müllerian-Derived Epithelial Cells
Source: Cancers (Basel). 2022 Feb 8;14(3):841. doi: 10.3390/cancers14030841 (PMC8834519; doi:10.3390/cancers14030841)
Supplement: Supplementary file 1 [file cancers-14-00841-s001.zip › cancers-1547238-supplementary.pdf]

Supplementary Figures and Figure Legends

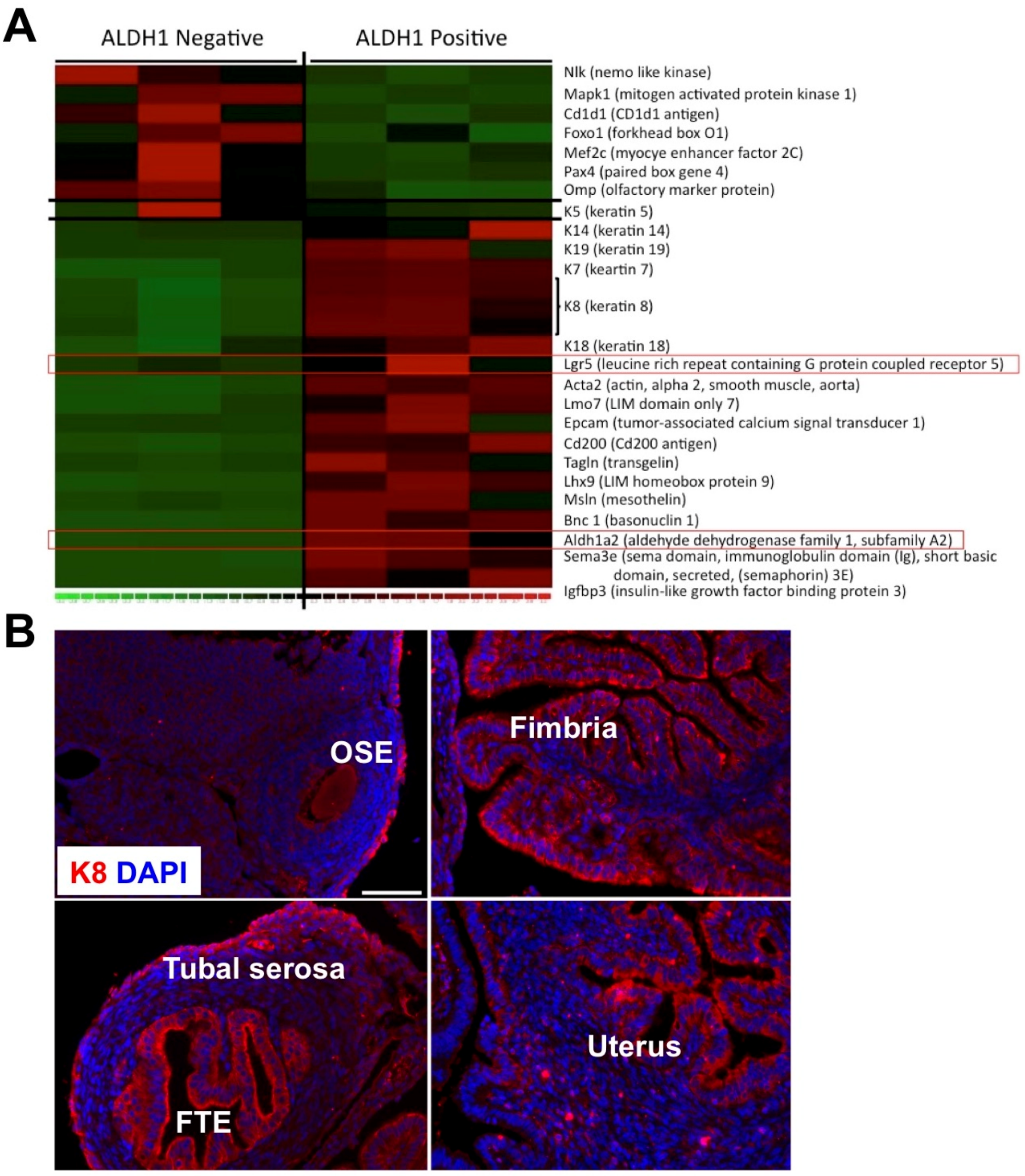

Figure S1. K8 expression in mesothelial cells and Müllerian epithelial cells. (A) Re-analysis of the publicly available microarray expression profiling data (based on Flesken-Nikitin et al., 2013). Select genes highly expressed in either ALDH$^+$ or ALDH$^-$ OSE cells are indicated. Red: higher expression; dark: intermediate expression; green: lower expression. The previously validated marker for ALDH$^+$ OSE stem cells, *Lgr5*, as well as one of the *ALDH* genes (*Aldh1a2*), are highlighted (i.e., higher expression in ALDH$^+$ cells) as the quality control for the microarray expression analysis. **(B)** Representative images of ovary, fallopian tube, and uterus from wildtype female mice showing Keratin 8 (K8) protein expression in ovarian surface epithelial cells (OSE) of ovary, mesothelial cells lining the tubal serosa, fimbrial and tubal epithelial cells (FTE) of fallopian tube (FT), and endometrial epithelial cells of uterus. Scale bar (applies to all pictures in the same Figure panel) = 50μm.

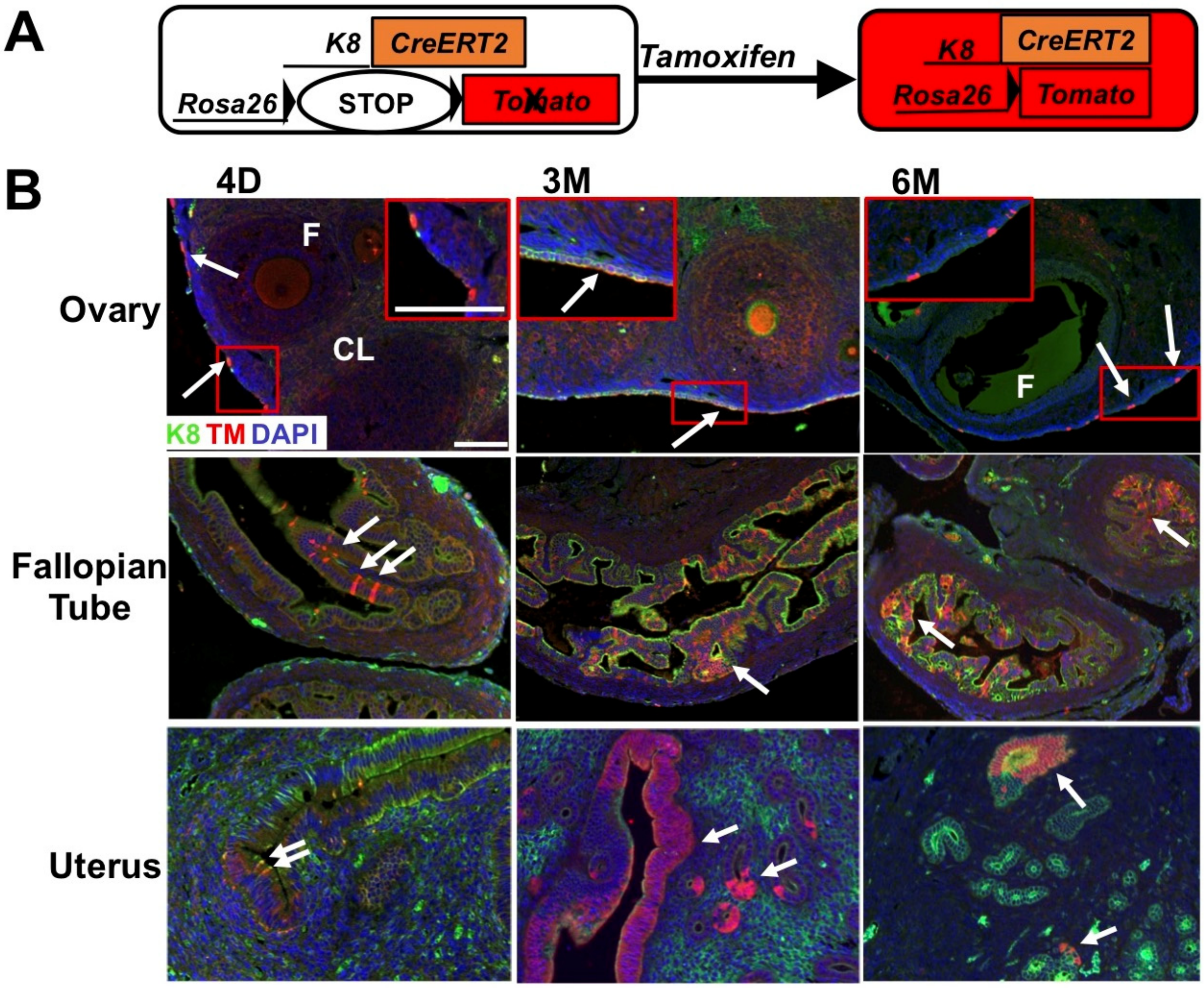

Figure S2. Tracing of the *Krt8*⁺ lineage. **(A)** Schematic diagram of *K8CreER*^{T2}*;R26tdTm* transgenic mouse model and activation of the Tomato reporter expression upon tamoxifen induction. **(B)** Representative images of ovary, fallopian tube and uterus from *K8-CreER*^{T2}*;R26tdTm* female mice after chasing for various time durations upon tamoxifen injection showing K8, Tomato (TM) and DAPI staining. Arrows indicate Tomato positive cells. 4D: 4 days; 3M: 3 months; 6M: 6 months; F: follicle; CL: Corpus luteum. Scale bar (applies to all pictures in the same Figure panel) = 50μm.

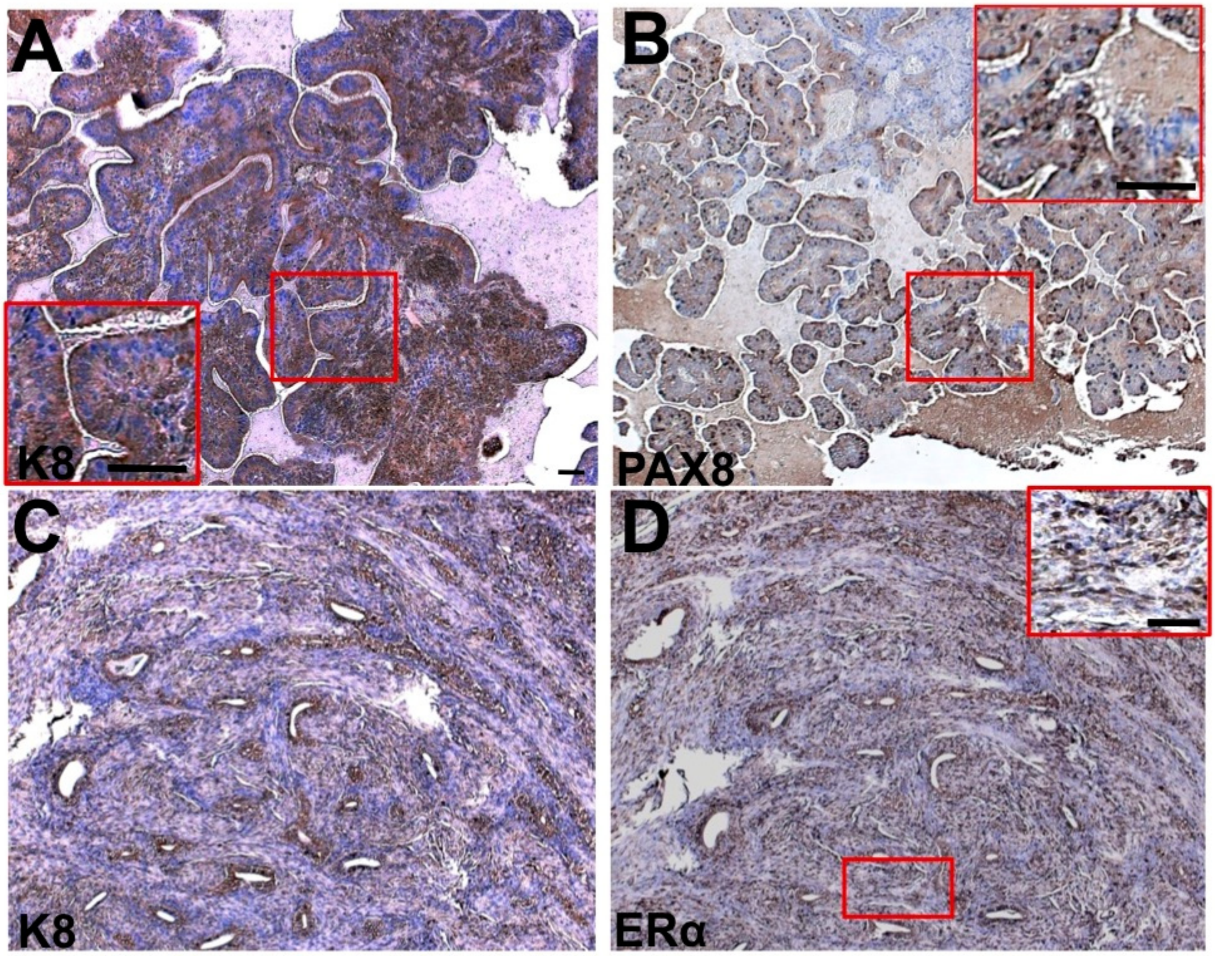

Figure S3. Immunohistochemical analyses of marker expression in gynecological malignancies developed in the p53 cohort. **(A,B)** Representative immunohistochemical images of indicated proteins from ovary of injected p53/Brca1 mice showing K8 (A) and PAX8 (B, inset showing nuclear staining) expression in the serous adenocarcinoma. **(C,D)** Representative immunohistochemical images of indicated proteins from uterus of injected p53/Rb1 mice showing K8 (C) and ER*α* (D) expression in the uterine endometrioid cancer. Scale bars = 50μm.

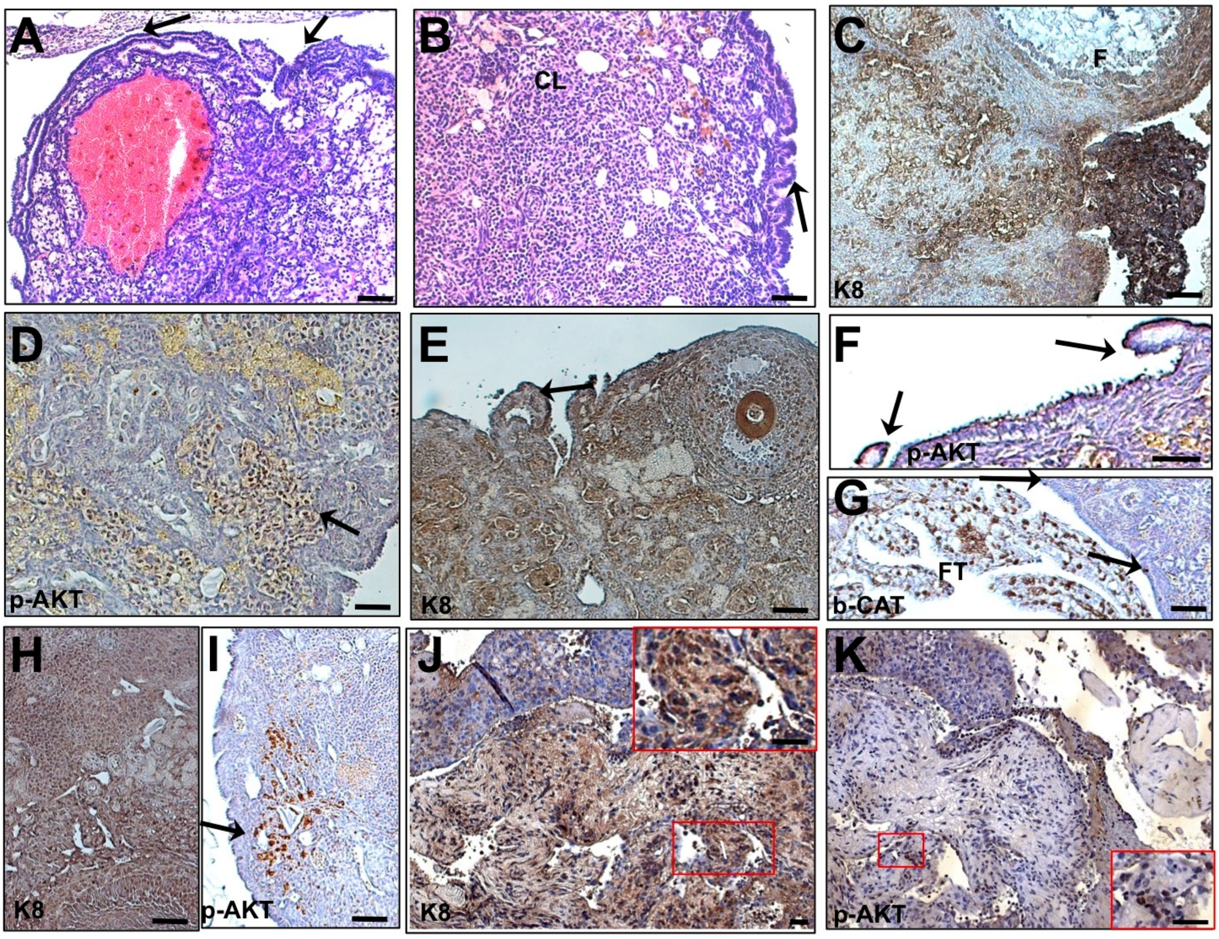

Figure S4. Histological and immunohistochemical analyses of gynecological malignancies developed in the Pten cohort. **(A-B)** Representative histological images showing endometrioid hyperplasia (A, arrows) and adenoma (B) in the ovary from the injected Pten/Dicer mice. **(C-K)** Representative immunohistochemical images showing expression of indicated protein markers from ovaries of the injected Pten/Kras (C and D), Pten/Apc (E-G), Pten/Dicer (H and I), and Pten/Pik3ca (J and K) mice. Arrows indicated positive staining of indicate markers in lesions. p-AKT: phosphorylated AKT; b-CAT: β-catenin; F: follicle; CL: Corpus luteum; FT: fallopian tube. Scale bars = 50μm.
